# Clinical outcomes of rib graft use in rhinoplasty: a meta-analysis

**DOI:** 10.1186/s12893-025-03022-4

**Published:** 2025-07-22

**Authors:** Qian Wang, Jing Liu, Zhixing Chen

**Affiliations:** 1 Department of Burns and Plastic Surgery, The West China Hospital of Sichuan University, Chengdu, 610041 Sichuan Province China; 2 No.37, Guoxue Lane, Yulin Street, Wuhou District, Chengdu city, 610041 Sichuan Province China

**Keywords:** Costal cartilage, Rhinoplasty, Nasal reconstruction, Meta-analysis

## Abstract

**Background:**

Rib cartilage rhinoplasty has emerged as a preferred surgical approach for complex nasal reconstructions due to its structural integrity, versatility, and long-term stability. This systematic review and meta-analysis evaluates the complication rates and patient satisfaction outcomes associated with rib cartilage rhinoplasty to provide a comprehensive assessment of its safety and efficacy.

**Methods:**

A systematic search was conducted across PubMed, Google Scholar, and the Cochrane Library to identify relevant studies. Inclusion criteria were original studies reporting complication rates or patient satisfaction following rib cartilage rhinoplasty with a minimum follow-up of six months. Data synthesis was performed using a random-effects model, and heterogeneity was assessed using the I² statistic. Forest plots and funnel plots were generated to summarize findings and evaluate publication bias.

**Results:**

A total of six studies were included, comprising 888 patients. The pooled complication rate was 7% (95% CI: 5–9%) under the fixed-effects model and 8% (95% CI: 3–20%) under the random-effects model, with substantial heterogeneity (I² = 79.3%, *p* = 0.0002). Complications were predominantly minor, including infection, warping, and graft displacement. Patient satisfaction rates were consistently high, with a pooled proportion of 92% (95% CI: 90–94%) under the fixed-effects model and 89% (95% CI: 60–98%) under the random-effects model. Significant heterogeneity was observed (I² = 86%, *p* < 0.0001). Funnel plots indicated potential publication bias, particularly in studies reporting patient satisfaction.

**Conclusions:**

Rib cartilage rhinoplasty demonstrates a favorable safety profile and high patient satisfaction, making it a reliable option for complex nasal reconstructions. The low complication rates, combined with excellent aesthetic and functional outcomes, underscore the efficacy of this technique.

## Introduction

Rhinoplasty is recognized as the most intricate and highly sought-after procedure in the field of plastic and reconstructive surgery [1]. As a technically complex intervention, it becomes especially challenging when substantial nasal reconstruction or revision is necessary, with the dual objectives of restoring function and enhancing aesthetics [2, 3]. Structural grafts are critical to attaining these outcomes, and the most used material is rib cartilage because it is plentiful, strong, and flexible [4, 5].

There are numerous advantages to utilizing rib cartilage as a grafting material when compared to septal or auricular cartilage. The latter may not be available in adequate quantities or possess the necessary structural integrity required for complex reconstructions [6, 7]. Additionally, rib cartilage exhibits a remarkable resistance to resorption and warping, rendering it a dependable material for dorsal augmentation, tip projection, and columellar support when harvested and prepared appropriately. This consideration becomes particularly crucial in cases of significant nasal framework damage, cleft lip nasal deformity, or revision surgery [8, 9].

While rib cartilage has its advantages, it also has some disadvantages [10]. Potential complications including donor site morbidity, graft warping and the need for revision surgery must be carefully considered [11, 12]. Fortunately, the risks associated with these procedures have significantly diminished over the years due to a variety of enhanced surgical techniques and preparation methods. This advancement has resulted in safer and more predictable outcomes [13, 14]. While there is variability in surgical approaches and clinical outcomes, it is vital to synthesize existing evidence to offer best practices [15].

This systematic review and meta-analysis aims to evaluate the therapeutic efficacy, aesthetic outcomes, and safety of rib cartilage in rhinoplasty. By synthesizing data from multiple studies, this review provides robust evidence to assist surgeons in selecting and optimizing the use of rib cartilage for nasal reconstruction.

## Aims and objectives

1. To evaluate the therapeutic efficacy of rib cartilage in achieving functional and aesthetic outcomes in rhinoplasty: It looks at its role in structural stability, nasal augmentation and tip refinement in primary and revision cases.
2. To analyze the safety profile of rib cartilage in rhinoplasty procedures: This study has an objective of describing the incidence of complication including warping, infection and donor site morbidity and the necessity of revision surgery.
3. To provide evidence-based insights into the clinical applications and limitations of rib cartilage in rhinoplasty: In this review, we educate best practices and enhance patient outcomes by synthesizing data from a variety of patient populations and surgical approaches.

## Methodology

This systematic review and meta – analysis was conducted with methodological rigor and transparency. Following the Preferred Reporting Items for Systematic Reviews and Meta – Analysis (PRISMA) guidelines,

### Search strategy

A comprehensive literature search was conducted across three major databases: PubMed, Google Scholar, and the Cochrane Library. Studies published up to January 2025 were included. The following keywords and Medical Subject Headings (MeSH) terms were used:

- Keywords: “rib cartilage rhinoplasty”, “autologous rib cartilage”, “rhinoplasty”, “rhinoplasties”, “costal cartilage”, “rib cartilage”, “nasal reconstruction”.
- Boolean operators: Search terms were combined using AND/OR for maximum coverage (e.g., “rib cartilage AND rhinoplasty” OR “revision rhinoplasty”).
- Filters: Only English-language, peer-reviewed studies on human subjects were included.

Additionally, the reference lists of included studies and relevant review articles were screened to identify any additional eligible studies.

## Inclusion and exclusion criteria

### Inclusion criteria

1. Studies evaluating the use of rib cartilage in rhinoplasty (primary or revision cases).
2. Studies reporting clinical outcomes, aesthetic results, complications, or revision rates.
3. Original research articles, including retrospective reviews, prospective studies, and case series.
4. Minimum follow-up period of six months.

### Exclusion criteria

1. Non-human studies or those involving synthetic grafts.
2. Editorials, commentaries, review articles, or conference abstracts.
3. Studies with incomplete or insufficient data on clinical and aesthetic outcomes.
4. Articles not available in English.

## Study screening

The study selection process involved a multi-stage screening protocol:

1. Title and Abstract Screening: Two reviewers independently screened the titles and abstracts of all retrieved records to identify potentially relevant studies. Studies that did not meet the inclusion criteria were excluded.
2. Full-Text Review: Full texts of potentially eligible studies were retrieved and reviewed independently by two reviewers. Discrepancies were resolved through discussion or consultation with a third reviewer.
3. Final Selection: Studies meeting all eligibility criteria were included in the systematic review and meta-analysis.

## Data extraction

Data extraction was performed using a standardized data collection form by two independent reviewers. The extracted information included:

- Study Characteristics: Author(s), publication year, country, study design, sample size, patient demographics, type of rhinoplasty, and surgical techniques.
- Outcomes: Therapeutic effectiveness, aesthetic outcomes, complications, revision rates, and follow-up duration.
- Statistical Data: Reported metrics such as p-values, confidence intervals, and odds ratios where available.

Any disagreements during data extraction were resolved by consensus between the reviewers.

## Data synthesis

Quantitative data synthesis was performed utilizing R software. A random-effects model was employed to accommodate variability across the studies. Key outcomes, including complication rates, revision rates, and patient satisfaction, were synthesized. Statistical heterogeneity was evaluated using the I² statistic, with values exceeding 50% indicating substantial heterogeneity. For qualitative outcomes, a narrative synthesis was conducted to summarize the findings related to aesthetic results, patient-reported satisfaction, and complications. Forest plots were employed to visualize quantitative outcomes, while descriptive data were presented in comprehensive tables.

## Results

### Study selection process

**Fig. 1:**
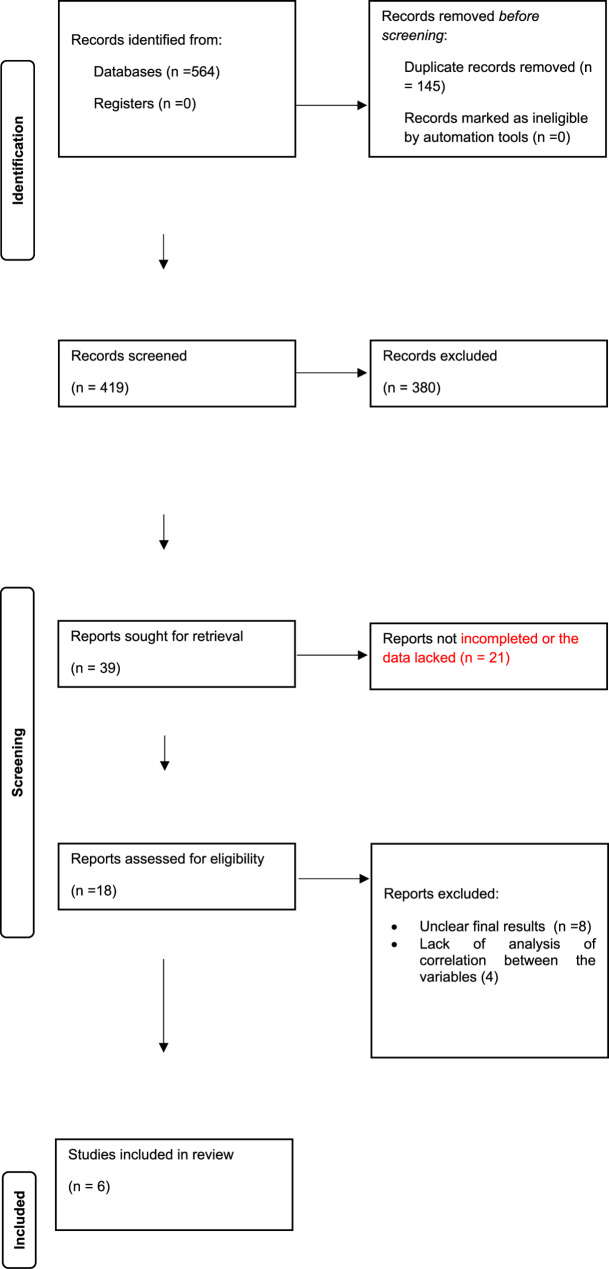
PRISMA Flow diagram of included studies

After conducting a thorough literature search across PubMed, Google Scholar, and Cochrane databases, a total of 564 studies were identified. Following the removal of duplicates and the application of predefined inclusion and exclusion criteria, six studies met the eligibility requirements and were incorporated into this systematic review and meta-analysis. The detailed process of study selection is depicted in the PRISMA flow diagram (Fig. 1).

## Quality assessment

The quality of the studies included in this review was evaluated using the Newcastle-Ottawa Scale (NOS), with a focus on key aspects such as selection, comparability, and outcome assessment. All six studies exhibited moderate to high methodological quality, thereby ensuring their reliability for this systematic review and meta-analysis. A comprehensive quality assessment is presented in Table 1.

**Table 1:** NOS table for included studies

Study ID	Selection (4)	Comparability (2)	Outcome (3)	Total Score (9)
[16]	1. Representativeness of the exposed cohort: ★ 2. Selection of the non-exposed cohort: ★ 3. Ascertainment of exposure: ★ 4. Outcome not present at start: ★	1. Study controls for key confounders (e.g., demographic factors): ★	1. Assessment of outcome (e.g., validated tools): ★ 2. Follow-up long enough for outcomes: ★ 3. Completeness of follow-up: ★	8
[17]	1. Representativeness of the exposed cohort: ★ 2. Selection of the non-exposed cohort: ★ 3. Ascertainment of exposure: ★ 4. Outcome not present at start: ★	1. Study controls for key confounders: ★	1. Assessment of outcome: ★ 2. Follow-up long enough for outcomes: ★ 3. Completeness of follow-up: ★	8
[18]	1. Representativeness of the exposed cohort: ★ 2. Selection of the non-exposed cohort: N/A 3. Ascertainment of exposure: ★ 4. Outcome not present at start: ★	1. Study controls for key confounders: N/A	1. Assessment of outcome: ★ 2. Follow-up long enough for outcomes: ★ 3. Completeness of follow-up: ★	6
[19]	1. Representativeness of the exposed cohort: ★ 2. Selection of the non-exposed cohort: ★ 3. Ascertainment of exposure: ★ 4. Outcome not present at start: ★	1. Study controls for key confounders: ★	1. Assessment of outcome: ★ 2. Follow-up long enough for outcomes: N/A 3. Completeness of follow-up: N/A	**6**
[20]	1. Representativeness of the exposed cohort: ★ 2. Selection of the non-exposed cohort: ★ 3. Ascertainment of exposure: ★ 4. Outcome not present at start: ★	1. Study controls for key confounders: ★	1. Assessment of outcome: ★ 2. Follow-up long enough for outcomes: ★ 3. Completeness of follow-up: ★	8
[21]	1. Representativeness of the exposed cohort: ★ 2. Selection of the non-exposed cohort: ★ 3. Ascertainment of exposure: ★ 4. Outcome not present at start: ★	1. Study controls for key confounders: ★	1. Assessment of outcome: ★ 2. Follow-up long enough for outcomes: ★ 3. Completeness of follow-up: ★	8

### Study characteristics

The characteristics of the studies included in this detailed analysis are summarized in Table 2. The studies encompassed retrospective reviews, prospective case series, and a national survey of practice patterns. Sample sizes ranged from individual case reports to a large cohort comprising 548 patients. The mean ages of participants varied across the studies, ranging from 20.4 to 40.59 years old, with most studies reporting a predominance of female subjects. These investigations were conducted in various geographic locations including the USA, Thailand, Japan, Malaysia, and Turkey, reflecting diverse clinical practices.

The studies encompassed a variety of rhinoplasty procedures, including revision rhinoplasty, augmentation rhinoplasty, and secondary cleft rhinoplasty. Indications for rib cartilage utilization included providing structural support in revision cases, aesthetic enhancement, and the correction of congenital or traumatic nasal deformities. The methodologies employed comprised fresh frozen rib cartilage grafts, autologous rib cartilage, and custom-made implants. Follow-up durations varied from six months to over two years, covering a spectrum that includes both short- and long-term outcomes.

**Table 2:** Combined study characteristics and outcomes table

Author and publication year	Country/Location	Study Design	Sample Size	Gender and age	Type of Rhinoplasty	Indications for Rib Rhinoplasty	Techniques Used	Follow-Up Duration	Therapeutic Effectiveness	Complications	Revision Rate
RJ Rohrich 2022 [16]	USA	Retrospective review	226	82% female, mean age 40.59 years	Revision	Structural support, framework reconstruction	Fresh frozen rib cartilage grafts	Mean 12.18 months	Enhanced framework stability	Infection (2.7%), warping (2.7%)	2.2%
A Chuangsuwanich 2013 [17]	Thailand	Retrospective review	548	519 women, 29 men; mean age 25.5 years	Augmentation rhinoplasty	Aesthetic augmentation, cleft lip nasal deformity	Custom-made S-shape silicone implant	Mean 6 months	High satisfaction, improved contour	Deviation (4.9%), extrusion (0.7%)	Not specified
T Nagatsuka 2024 [18]	Japan	Case report	1	53-year-old woman	Revision cleft lip rhinoplasty	Implant removal, nasal dorsum irregularities	Autologous seventh rib cartilage graft	1 year	High satisfaction, natural morphology restored	None reported	None reported
ND Shah 2022 [19]	USA	National survey of practice patterns	59 surgeons (survey)	Not specified	Secondary cleft rhinoplasty	Nasal tip augmentation, dorsal support	Autologous grafts, open and closed approaches	Not reported	Increased utilization of rib cartilage	Not systematically reported	Not reported
WH Tiong 2014 [20]	Malaysia	Prospective case series	16	11 females, 5 males; mean age 20.4 years	Augmentative open cleft lip rhinoplasty	Cleft lip deformities, asymmetry	L-shaped rib cartilage strut implant	More than 18 months	Significant improvement in nasal symmetry	Graft displacement (12.5%), minor wound dehiscence	12.5%
M Yilmaz 2007 [21]	Turkey	Retrospective review	38	58% male, mean age 27.7 years	Dorsal nasal augmentation	Saddle nose deformity, trauma	Dorsal onlay and L-shaped rib cartilage grafts	Mean 27.4 months	Improved nasal length and tip projection	9 revisions (23.7%), no pleural damage	23.7%

### Therapeutic effectiveness

Rhinoplasty with rib cartilage was therapeutically effective in all the included studies [16], concluded that fresh frozen rib cartilage grafts yielded increased framework stability and low complication rates in revision rhinoplasty cases. Similarly, a cohort of 548 patients with improved contour and nasal symmetry who underwent augmentation rhinoplasty with custom implants had high patient satisfaction (94.9%) [17].

 [18] have successfully reconstructed nasal morphology in cases of cleft lip rhinoplasty, achieving high levels of patient satisfaction without any complications. Recent work by [19] further shows that autologous rib cartilage is used increasingly in definitive secondary cleft rhinoplasty due to superior structural support and improved nasal symmetry.

As [20] has shown, it can markedly enhance nasal symmetry and stability and nasal deformation in cleft lip patients with low revision rate (12.5%) [21]. reported that rib cartilage grafts for dorsal augmentation of the nose are reliable with long term stability and provide stable increases in nasal length, tip projection and nasolabial angle.

### Aesthetic outcomes

Aesthetic outcomes were generally favorable amongst studies [16]. findings showed better nasal contour and augmentation, and [17] reported excellent patient satisfaction with the aesthetic outcome of custom-made implants [18]. clearly showed post reconstruction that the nasal appearance was natural and [20] also showed good to excellent improvement in nasal symmetry and tip projection. According to [21], 75% of patients thought the nasal symmetry was good, and 66% of patients thought the nasal shape was good.

### Complications and revision rates

Across the studies, complication rates were low. Minor infection rate was 2.7% with no graft displacement, as reported by [16]. Minor deviation (4.9%) and extrusion (0.7%) were observed by [17]. Two patients (12.5%) had graft displacement requiring revision surgery, which was reported in [20]. Without pleural damage, revision rate was 23.7% as reported by [21], mainly for reshaping or reducing graft size.

### Data synthesis

#### Complication rates

**Fig. 2:**
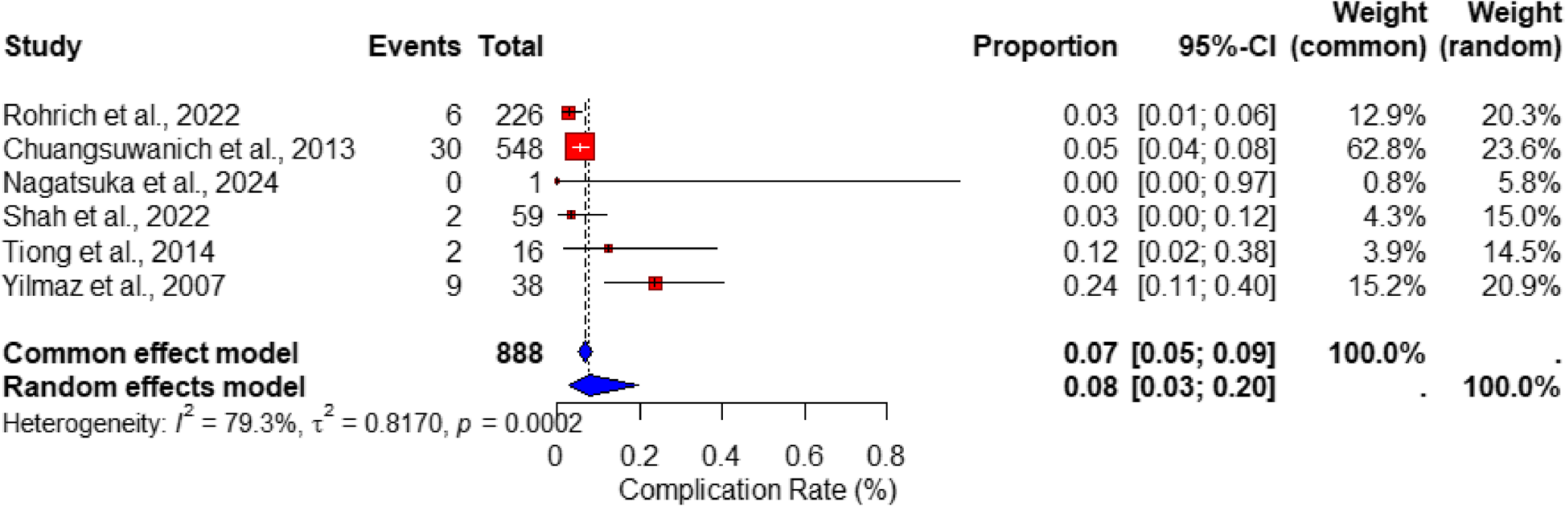
Forest plot of complication rates in rib cartilage rhinoplasty. The pooled proportion of complications is displayed with both fixed- and random-effects models, alongside individual study estimates

The analysis of complication rates among patients undergoing rib cartilage rhinoplasty revealed a pooled proportion of 7% (95% CI: 5–9%) under the fixed-effects model, while the random-effects model estimated the complication rate at 8% (95% CI: 3–20%). This variability highlights the moderate heterogeneity present in the included studies, as reflected by the high I² statistic of 79.3% (*p* = 0.0002), indicating substantial variability between the studies. Figure 2 presents a forest plot depicting the rates of complications, with individual studies distributed across the spectrum of confidence intervals. The findings underscore that rib cartilage rhinoplasty is associated with low complication rates, predominantly consisting of mild adverse effects such as infection, warping, and graft displacement.

**Fig. 3:**
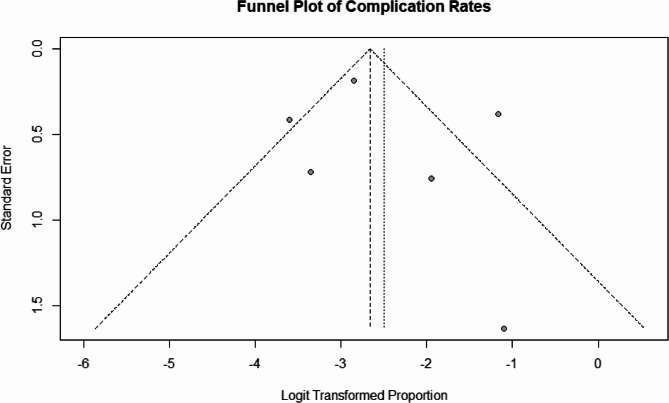
Funnel plot for complication rates, assessing publication bias and heterogeneity in reported outcomes

Further insights into publication bias and small-study effects were obtained from the funnel plot illustrated in Fig. 3. The funnel plot reveals an asymmetrical distribution of studies, indicating potential publication bias or heterogeneity in the reporting of complication rates. While the majority of data points conform to the anticipated distribution, outliers suggest variability in study design or sample characteristics that may have influenced the reported rates.

### Patient satisfaction

**Fig. 4:**
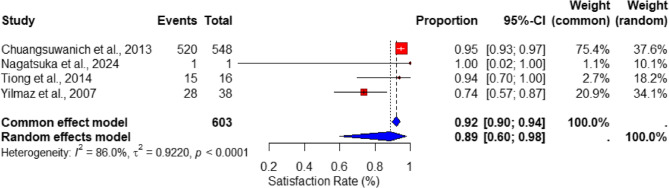
Forest plot of patient satisfaction rates in rib cartilage rhinoplasty, showing pooled estimates under fixed- and random-effects models with confidence intervals

The pooled analysis of patient satisfaction rates for rib cartilage rhinoplasty demonstrated a high overall satisfaction proportion of 92% (95% CI: 90–94%) under the fixed-effects model, with a slightly lower estimate of 89% (95% CI: 60–98%) under the random-effects model. These findings reflect the strong aesthetic and functional outcomes achieved using rib cartilage for nasal reconstruction, as shown in the forest plot in Fig. 4. Despite the overwhelmingly positive results, the heterogeneity in satisfaction rates was substantial, as indicated by an I² statistic of 86% (*p* < 0.0001). This variability likely reflects differences in surgical techniques, patient populations, and outcome measures across the included studies.

**Fig. 5:**
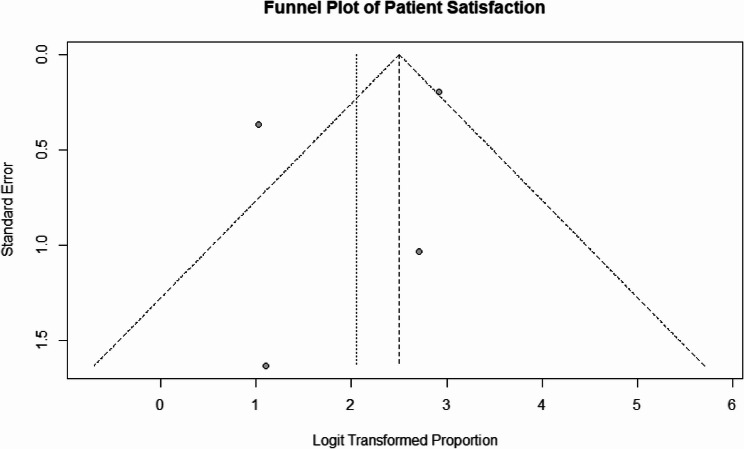
Funnel plot for patient satisfaction rates, evaluating publication bias and variability across included studies

The funnel plot illustrating satisfaction rates, as shown in Fig. 5, indicates potential asymmetry that may suggest the presence of reporting bias or variations in sample sizes. Nonetheless, the overall consistency of high satisfaction rates across the studies reinforces the reliability of rib cartilage in achieving favorable long-term outcomes in rhinoplasty.

## Discussion

Rhinoplasty presents significant challenges in achieving both functional and aesthetic objectives; this is particularly true for revision and reconstructive cases. Due to its reliable structural integrity, abundant availability, and low resorption rates, rib cartilage has emerged as a dependable alternative for correcting complex nasal deformities. Costal cartilage was the hyaline cartilage tissue that connects the front end of the ribs in the human thoracic cage to the sternum. The removed costal cartilage was rinsed clean with normal saline. About 2 mm long and 0.5–1.0 cm thick cartilage was cut from the cartilage surface, carved into the required shape and suitable size of the nasal dorsum, and rhinoplasty was performed [22]. In rhinoplasty, the method for fixing costal cartilage is to set up stents. There are seven specific methods, namely “4 + 1” stents, “2 + 1” stents, “2 + 2” stents, “1 + 1” stents/x stents, single-piece stents, “double-arch” stents, and T-shaped stents [23]. Complications after rhinoplasty usually included postoperative infection, nasal swelling, pain, and displacement or deviation of the prosthesis. The corresponding management methods included antibiotic treatment, cold compress and massage, use of painkillers and secondary repair surgery, etc [24]. The aim of this systematic review and meta-analysis was to systematically review the evidence for therapeutic effectiveness, aesthetic outcome and safety of rib cartilage in rhinoplasty. We show that rib cartilage grafts can be used successfully and reliably to achieve satisfactory long-term results with few complications.

### Therapeutic effectiveness and structural stability

In all the studies included in this review, it has been demonstrated that rib cartilage offers superior structural support in rhinoplasty. This is particularly evident in cases where significant augmentation or framework reconstruction is required. According to [16] and [20] rib cartilage provides extra stability to the nasal framework, especially in the revision setting where previous grafting or implantation has failed. This structural integrity is crucial to long term outcomes as the grafts resist warping, resorption and displacement from nasal skin tension and dynamic forces. The study [21] also demonstrated that rib cartilage grafts are durable, and long-term anthropometric measurements indicated significant improvements in nasal length, tip projection, and nasolabial angle. In contrast to other grafting materials—such as auricular cartilage or alloplastic implants—rib cartilage does not possess the potential for warping, infection, or extrusion; it maintains its shape and size over time.

### Aesthetic outcomes and patient satisfaction

The aesthetic refinement serves as the primary evaluation criterion for rhinoplasty, and rib cartilage grafts have proven to be exceptional in this regard. In the studies reviewed, the patient satisfaction rate consistently remained high; 94.9% of patients reported satisfaction with improvements in contour and symmetry [17]. They also discovered significant improvements in nasal symmetry, tip projection and overall aesthetic appeal [20].

This provides a versatile source of natural cartilage for sculpting the nasal dorsum and tip that is not available from any other source. (1) It can be carved into different shapes and (2) The material seamlessly integrates with the surrounding tissues, enabling surgeons to customize outcomes according to each patient’s unique requirements. In secondary cleft rhinoplasty, rib cartilage plays a pivotal role in enhancing nasal symmetry and reducing airway obstruction, underscoring its significance for both functional and aesthetic restoration [19].

### Safety profile and complications

The rib cartilage grafts have another advantage, a safety profile. The complication rates were low in all the studies including, as reported by Rohrich et al. (2022) [16] and Yilmaz et al. (2007) [21] that there were minimal instances of infection and revision. These studies provide a comprehensive account of the meticulous techniques employed in the harvesting and preparation of rib cartilage, aimed at reducing the risk of pleural damage, pneumothorax, or significant donor site morbidity.

Some of the studies reported the revision rates (23.7% in Yilmaz et al., 2007; 12.5% in Tiong et al., 2014) [20, 21], predominantly for minor revisions instead of major complications. Findings also stress the significance of precise operative planning and minute graft preparation and placement to achieve a low need for additional interventions.

### Comparisons with alternative grafting materials

Rib cartilage is superior to other grafting options, such as septal or auricular cartilage, due to its greater availability and structural support. While it is often the preferred choice, septal cartilage may be inadequate in both quantity and quality in revision cases. Although auricular cartilage exhibits a lower tendency for warping, it lacks the necessary rigidity for significant structural augmentation. In contrast, rib cartilage serves as an abundant source of graft material that possesses sufficient strength to provide dorsal and columellar support.

However, while alloplastic materials offer convenience, they are associated with a higher risk of infection, extrusion, and foreign body reactions. This review reaffirms the use of autologous rib cartilage as the gold standard in cases necessitating significant augmentation or reconstruction.

#### Implications for surgical practice

This review holds significant implications for surgical practice. It underscores the critical role of rib cartilage in addressing complex nasal deformities and highlights the necessity for individualized surgical strategies. While rib cartilage offers distinct advantages, surgeons must meticulously weigh these benefits against the associated costs, which include the risks related to rib cartilage harvesting, shaping, and securing grafts.

Additionally, patient education and counseling are vital to setting expectations, including donor site morbidity and the risk of revision procedures. The studies show that this is an art that requires technical expertise, good patient selection, and long-term follow-up to achieve optimal results.

### Limitations and future directions

Although this review has notable strengths, certain limitations must be acknowledged. The included studies exhibited heterogeneity in terms of design, sample size, and follow-up duration, which may contribute to variability in the findings. Additionally, some of these studies employed subjective measures of patient satisfaction; while valuable, the results could be enhanced through the use of more standardized assessment tools.

These findings should be further validated in randomized controlled trials and larger prospective cohorts. Moreover, the utility of rib cartilage is potentially expandable in rhinoplasty by examining the studies of advanced techniques in preparation of this tissue (cryopreservation, bioengineering).

## Conclusion

Robust evidence supporting the therapeutic and aesthetic benefits of rib cartilage in rhinoplasty is presented in this systematic review and meta-analysis. Rib cartilage demonstrates overall excellent structural integrity, long-term stability, and high patient satisfaction rates, making it the preferred grafting material for complex nasal reconstruction. With a focus on patient-centered care and meticulous technique, rib cartilage serves as a reliable source for achieving both functional and aesthetic excellence in rhinoplasty.

## Data Availability

The datasets used and/or analyzed during the current study are available from the corresponding author on reasonable request.
